# Prizegiving at the Middlesex Hospital

**Published:** 1923-11

**Authors:** 


### PRIZE-GIVING AT MIDDLESEX HOSPITAL.

THE opening of the Winter Session of the Middlesex Hospital Medical School and the distribution of prizes by Princess Alice, took place at the Scala Theatre on October 2, and was a very successful function. The school is growing rapidly in importance and efficiency and seems to have before it a future of ever-increasing uesfulness. There was, thus, a general air of enthusiasm and buoyancy which made the proceedings particularly pleasant. As usual, the first item was a brief address on some new phase of scientific research, and Mr. Somerville Hastings's paper on "Team Work in Nature" illustrated with lantern slides, while distinctly technical, was always lucid and interesting. In the medical profession the value of team work had, he said, been recognised from the earliest times. When an engineer finds out a new process he patents it, so that none but he may use it. When a doctor makes a discovery it is the tradition that he should publish it to the world, so that all his colleagues and their patients may benefit thereby. Mr. Webb-Johnson, the Dean, then gave his report on the work of the past year. He spoke of the distinction conferred on his alma mater by the election of Sir John Bland-Sutton as President of the Royal College of Surgeons. He also referred to the valuable step that had been taken by the provision of new wards for dealing with mental cases on the border line and to the developments in the Bland-Sutton Institute.

### A Critical Moment.

The distribution of prizes was held on the stage, on which were seated the College professors, imposing in their many-coloured hoods. It was followed by votes of thanks to Princess Alice and to the Earl of Athlone, who presided. Lord Athlone said that they all had just cause to be proud of their hospital and its school. He was casting no reflection upon Mr. Webb-Johnson's predecessors, of whose work they had lasting evidence, when he asserted that Mr. Webb-Johnson would be remembered by Middlesex men for generations to come as the man who, at a critical stage of the school's history, conceived and carried out schemes for its development which its founder, Sir Charles Bell, in his most imaginative mood, could hardly have thought to be possible. With certain ideals before him he worked unceasingly to realise them, and that he had succeeded, even beyond the expectations of those who knew him best, was gratefully admitted by all competent judges. Visitors then went over to the hospital, where tea was served, and parties were conducted round the school and research departments, including the new laboratories in the Bland-Sutton Institute.

